# (*E*)*-N*’-(2,3,4-Trimethoxy­benzyl­idene)isonicotinohydrazide

**DOI:** 10.1107/S1600536810015266

**Published:** 2010-04-30

**Authors:** H. S. Naveenkumar, Amirin Sadikun, Pazilah Ibrahim, Chin Sing Yeap, Hoong-Kun Fun

**Affiliations:** a School of Pharmaceutical Sciences, Universiti Sains Malaysia, 11800 USM, Penang, Malaysia; b X-ray Crystallography Unit, School of Physics, Universiti Sains Malaysia, 11800 USM, Penang, Malaysia

## Abstract

In the title compound, C_16_H_17_N_3_O_4_, the mol­ecule exists in an *E* configuration with respect to the C=N double bond. The mol­ecule is not planar, the dihedral angle between the pyridine and benzene rings being 71.67 (8)°. In the crystal structure, mol­ecules are linked into chains along the *b* axis by bifurcated N—H⋯O and C—H⋯O hydrogen bonds. These chains are linked into a three-dimensional network by C—H⋯O and C—H⋯π inter­actions.

## Related literature

For applications of isoniazid derivatives, see: Janin (2007 ▶); Maccari *et al.* (2005 ▶); Slayden & Barry (2000 ▶); Kahwa *et al.* (1986 ▶). For preparation of the compound, see: Lourenco *et al.* (2008 ▶). For related structures, see: Naveenkumar *et al.* (2009 ▶, 2010*a* ▶,*b* ▶); Shi (2005 ▶). For the stability of the temperature controller used for the data collection, see: Cosier & Glazer (1986 ▶).
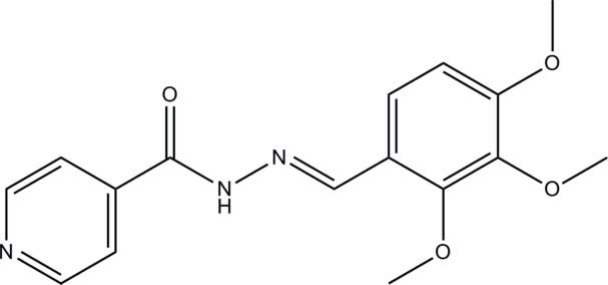


## Experimental

### 

#### Crystal data

C_16_H_17_N_3_O_4_
*M* *_r_* = 315.33
Monoclinic, 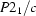

*a* = 14.246 (3) Å
*b* = 9.397 (2) Å
*c* = 12.098 (3) Å
β = 109.245 (6)°
*V* = 1529.1 (6) Å^3^
*Z* = 4
Mo *K*α radiation
μ = 0.10 mm^−1^
*T* = 100 K
0.38 × 0.33 × 0.14 mm

#### Data collection

Bruker APEXII DUO CCD area-detector diffractometer
Absorption correction: multi-scan (*SADABS*; Bruker, 2009 ▶) *T*_min_ = 0.963, *T*_max_ = 0.986
14341 measured reflections
3482 independent reflections
2988 reflections with *I* > 2σ(*I*)
*R*_int_ = 0.030

#### Refinement

*R*[*F* ^2^ > 2σ(*F* ^2^)] = 0.047
*wR*(*F* ^2^) = 0.159
*S* = 1.10
3482 reflections
215 parameters
H atoms treated by a mixture of independent and constrained refinement
Δρ_max_ = 0.57 e Å^−3^
Δρ_min_ = −0.38 e Å^−3^

### 

Data collection: *APEX2* (Bruker, 2009 ▶); cell refinement: *SAINT* (Bruker, 2009 ▶); data reduction: *SAINT*; program(s) used to solve structure: *SHELXTL* (Sheldrick, 2008 ▶); program(s) used to refine structure: *SHELXTL*; molecular graphics: *SHELXTL*; software used to prepare material for publication: *SHELXTL* and *PLATON* (Spek, 2009 ▶).

## Supplementary Material

Crystal structure: contains datablocks global, I. DOI: 10.1107/S1600536810015266/tk2657sup1.cif

Structure factors: contains datablocks I. DOI: 10.1107/S1600536810015266/tk2657Isup2.hkl

Additional supplementary materials:  crystallographic information; 3D view; checkCIF report

## Figures and Tables

**Table 1:** Hydrogen-bond geometry (Å, °) *Cg*1 is the centroid of the C1/C2/N1/C3/C4/C5 ring.

*D*—H⋯*A*	*D*—H	H⋯*A*	*D*⋯*A*	*D*—H⋯*A*
N2—H1*N*2⋯O1^i^	0.91 (3)	2.09 (3)	2.7987 (19)	134 (2)
C4—H4*A*⋯O1^i^	0.93	2.46	3.348 (2)	159
C14—H14*C*⋯O3	0.96	2.55	3.113 (2)	118
C15—H15*C*⋯O2^ii^	0.96	2.49	3.292 (2)	141
C16—H16*A*⋯O3^iii^	0.96	2.57	3.518 (2)	167
C14—H14*B*⋯*Cg*1^i^	0.96	2.81	3.719 (3)	159

Symmetry codes: (i) 
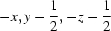
; (ii) 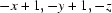; (iii) 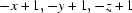.

**Fig. 1.**
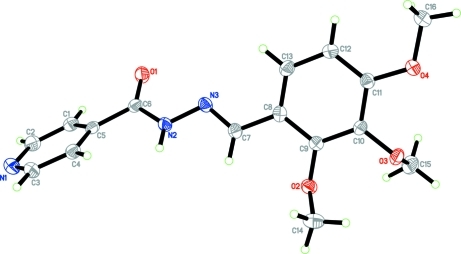
The molecular structure of (I) with atom labels and 50% probability ellipsoids for non-H atoms.

**Fig. 2.**
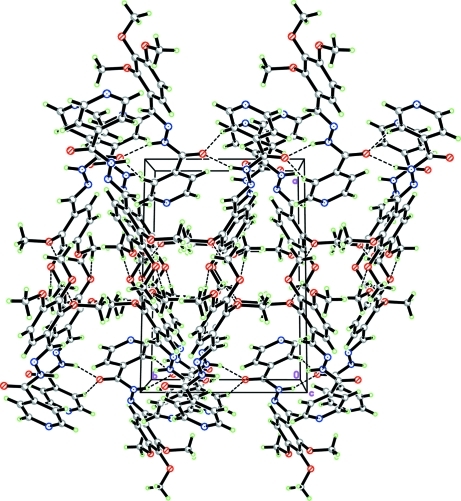
The crystal packing of (I), viewed down the c axis, showing the molecules are linked into a 3-D network. Intermolecular contacts are shown as dashed lines.

## supplementary crystallographic information

### Comment

In the search of new compounds of pharmaceutical importance, isoniazid derivatives have been found to possess potential tuberculostatic activity (Janin, 2007; Maccari *et al.*, 2005; Slayden & Barry, 2000). Schiff bases have attracted much attention because of their biological activity (Kahwa *et al.*, 1986). As a part of on-going work into the synthesis of (*E*)-*N*'-substituted isonicotinohydrazide derivatives, in this paper we present the crystal structure of the title compound (I).

The geometric parameters of (I) are comparable to those in related structures (Naveenkumar *et al.*, 2009, 2010a, 2010b; Shi, 2005). The molecule exists in an *E* configuration with respect to the C7═N3 double bond (Fig. 1). The isoniazid group is twisted away with the torsion angle of C1–C5–C6–N2 being -149.60 (15)°. The dihedral angle between the pyridine ring and the benzene ring is 71.67 (8)°. One of the methoxy group is coplanar with the benzene ring whereas the other two are twisted away from the benzene ring [torsion angles: C16–O4–C11–C12 = -3.8 (2), C15–O3–C10–C11 = -97.83 (17), C14–O2–C9–C10 = 73.23 (19) °]. A weak intramolecular C14–H14C···O3 hydrogen bond stabilizes the molecular structure (Table 1). In the crystal structure, the molecules are linked into one-dimensional chains along the *b* axis by the bifurcated intermolecular N2–H1N2···O1 and C4–H4A···O1 hydrogen bonds (Table 1). These chains are linked into a three-dimensional network by C15–H15C···O2, C16–H16A···O3 and C14–H14B···*Cg*1 interactions (Fig. 2, Table 1).

### Experimental

The isoniazid derivative (I) was prepared following the procedure by Lourenco *et al.* (2008). 2,3,4-Trimethoxybenzaldehyde (1.0 eq) was reacted with isoniazid (1.0 eq) in ethanol/water. After stirring for 3 h at room temperature, the resulting mixture was concentrated under reduced pressure. The residue was purified by washing with cold ethanol and ethyl ether, affording the pure derivative. The colourless crystals were obtained by recrystallization from a methanol solution of (I).

### Refinement

The H1N2 H atom was located from a difference Fourier map and refined freely. The remaining H atoms were positioned geometrically [C–H = 0.93 or 0.96 Å] and refined using a riding model, with *U*_iso_(H) = 1.2 or 1.5 *U*_eq_(C). A rotating-group model was applied for the methyl groups.

### Crystal data

**Table:**

C_16_H_17_N_3_O_4_	*F*(000) = 664
*M**_r_* = 315.33	*D*_x_ = 1.370 Mg m^−^^3^
Monoclinic, *P*2_1_/*c*	Mo *K*α radiation, λ = 0.71073 Å
Hall symbol: -P 2ybc	Cell parameters from 5847 reflections
*a* = 14.246 (3) Å	θ = 2.8–33.0°
*b* = 9.397 (2) Å	µ = 0.10 mm^−^^1^
*c* = 12.098 (3) Å	*T* = 100 K
β = 109.245 (6)°	Block, colourless
*V* = 1529.1 (6) Å^3^	0.38 × 0.33 × 0.14 mm
*Z* = 4	

### Data collection

**Table:**

Bruker APEXII DUO CCD area-detector diffractometer	3482 independent reflections
Radiation source: fine-focus sealed tube	2988 reflections with *I* > 2σ(*I*)
graphite	*R*_int_ = 0.030
φ and ω scans	θ_max_ = 27.5°, θ_min_ = 2.6°
Absorption correction: multi-scan (*SADABS*; Bruker, 2009)	*h* = −17→18
*T*_min_ = 0.963, *T*_max_ = 0.986	*k* = −11→12
14341 measured reflections	*l* = −15→15

### Refinement

**Table:**

Refinement on *F*^2^	Primary atom site location: structure-invariant direct methods
Least-squares matrix: full	Secondary atom site location: difference Fourier map
*R*[*F*^2^ > 2σ(*F*^2^)] = 0.047	Hydrogen site location: inferred from neighbouring sites
*wR*(*F*^2^) = 0.159	H atoms treated by a mixture of independent and constrained refinement
*S* = 1.10	*w* = 1/[σ^2^(*F*_o_^2^) + (0.0903*P*)^2^ + 0.7464*P*] where *P* = (*F*_o_^2^ + 2*F*_c_^2^)/3
3482 reflections	(Δ/σ)_max_ < 0.001
215 parameters	Δρ_max_ = 0.56 e Å^−^^3^
0 restraints	Δρ_min_ = −0.38 e Å^−^^3^

### Special details

**Table:**

Experimental. The crystal was placed in the cold stream of an Oxford Cryosystems Cobra open-flow nitrogen cryostat (Cosier & Glazer, 1986) operating at 100.0 (1) K.
Geometry. All esds (except the esd in the dihedral angle between two l.s. planes) are estimated using the full covariance matrix. The cell esds are taken into account individually in the estimation of esds in distances, angles and torsion angles; correlations between esds in cell parameters are only used when they are defined by crystal symmetry. An approximate (isotropic) treatment of cell esds is used for estimating esds involving l.s. planes.
Refinement. Refinement of F^2^ against ALL reflections. The weighted R-factor wR and goodness of fit S are based on F^2^, conventional R-factors R are based on F, with F set to zero for negative F^2^. The threshold expression of F^2^ > 2sigma(F^2^) is used only for calculating R-factors(gt) etc. and is not relevant to the choice of reflections for refinement. R-factors based on F^2^ are statistically about twice as large as those based on F, and R- factors based on ALL data will be even larger.

### Fractional atomic coordinates and isotropic or equivalent isotropic displacement parameters (Å^2^)

**Table:**

	*x*	*y*	*z*	*U*_iso_*/*U*_eq_	
O1	0.01090 (9)	0.86823 (12)	−0.28320 (11)	0.0286 (3)	
O2	0.36424 (9)	0.41851 (12)	−0.00731 (10)	0.0232 (3)	
O3	0.47223 (8)	0.40332 (13)	0.23303 (10)	0.0235 (3)	
O4	0.41436 (9)	0.55428 (13)	0.38640 (10)	0.0245 (3)	
N1	−0.18863 (12)	0.63126 (17)	−0.66119 (13)	0.0302 (4)	
N2	0.05989 (10)	0.63755 (14)	−0.24901 (12)	0.0207 (3)	
N3	0.11895 (10)	0.66421 (14)	−0.13400 (11)	0.0208 (3)	
C1	−0.08316 (12)	0.79807 (18)	−0.52380 (15)	0.0238 (3)	
H1A	−0.0557	0.8888	−0.5098	0.029*	
C2	−0.14580 (13)	0.76010 (19)	−0.63502 (15)	0.0277 (4)	
H2A	−0.1587	0.8275	−0.6944	0.033*	
C3	−0.16847 (13)	0.53782 (19)	−0.57329 (15)	0.0278 (4)	
H3A	−0.1983	0.4487	−0.5894	0.033*	
C4	−0.10626 (12)	0.56370 (17)	−0.45947 (15)	0.0231 (3)	
H4A	−0.0942	0.4937	−0.4021	0.028*	
C5	−0.06239 (11)	0.69824 (17)	−0.43404 (14)	0.0204 (3)	
C6	0.00512 (11)	0.74314 (16)	−0.31422 (14)	0.0203 (3)	
C7	0.18304 (11)	0.56473 (17)	−0.09093 (13)	0.0200 (3)	
H7A	0.1910	0.4914	−0.1387	0.024*	
C8	0.24315 (11)	0.56734 (16)	0.03316 (14)	0.0198 (3)	
C9	0.33100 (11)	0.48599 (16)	0.07346 (13)	0.0184 (3)	
C10	0.38660 (11)	0.48299 (16)	0.19251 (13)	0.0188 (3)	
C11	0.35435 (12)	0.56152 (17)	0.27246 (14)	0.0205 (3)	
C12	0.26610 (12)	0.63962 (17)	0.23309 (15)	0.0233 (3)	
H12A	0.2438	0.6895	0.2860	0.028*	
C13	0.21207 (12)	0.64218 (17)	0.11454 (14)	0.0229 (3)	
H13A	0.1537	0.6951	0.0886	0.027*	
C14	0.35843 (15)	0.26623 (19)	−0.00823 (17)	0.0325 (4)	
H14A	0.3768	0.2297	−0.0723	0.049*	
H14B	0.2917	0.2376	−0.0171	0.049*	
H14C	0.4029	0.2294	0.0641	0.049*	
C15	0.55771 (13)	0.4749 (2)	0.22131 (17)	0.0324 (4)	
H15A	0.6152	0.4152	0.2516	0.049*	
H15B	0.5682	0.5626	0.2644	0.049*	
H15C	0.5468	0.4945	0.1401	0.049*	
C16	0.38727 (14)	0.6398 (2)	0.46951 (15)	0.0308 (4)	
H16A	0.4343	0.6248	0.5463	0.046*	
H16B	0.3221	0.6132	0.4690	0.046*	
H16C	0.3873	0.7384	0.4488	0.046*	
H1N2	0.0529 (18)	0.544 (3)	−0.270 (2)	0.038 (6)*	

### Atomic displacement parameters (Å^2^)

**Table:**

	*U*^11^	*U*^22^	*U*^33^	*U*^12^	*U*^13^	*U*^23^
O1	0.0297 (6)	0.0180 (6)	0.0300 (6)	0.0029 (5)	−0.0011 (5)	−0.0029 (5)
O2	0.0270 (6)	0.0225 (6)	0.0204 (6)	0.0008 (4)	0.0083 (5)	−0.0008 (4)
O3	0.0192 (6)	0.0264 (6)	0.0232 (6)	0.0059 (4)	0.0047 (4)	0.0030 (4)
O4	0.0264 (6)	0.0278 (6)	0.0176 (6)	0.0044 (5)	0.0048 (5)	−0.0011 (4)
N1	0.0324 (8)	0.0321 (8)	0.0239 (7)	0.0010 (6)	0.0062 (6)	−0.0048 (6)
N2	0.0224 (7)	0.0167 (6)	0.0202 (6)	0.0005 (5)	0.0032 (5)	−0.0022 (5)
N3	0.0217 (6)	0.0189 (6)	0.0200 (6)	−0.0027 (5)	0.0045 (5)	−0.0024 (5)
C1	0.0234 (7)	0.0205 (8)	0.0288 (8)	0.0018 (6)	0.0104 (6)	0.0013 (6)
C2	0.0306 (9)	0.0289 (9)	0.0244 (8)	0.0040 (7)	0.0101 (7)	0.0026 (6)
C3	0.0276 (8)	0.0244 (8)	0.0285 (8)	−0.0007 (7)	0.0055 (7)	−0.0062 (7)
C4	0.0214 (7)	0.0200 (8)	0.0272 (8)	0.0014 (6)	0.0071 (6)	0.0000 (6)
C5	0.0181 (7)	0.0197 (7)	0.0243 (8)	0.0031 (6)	0.0080 (6)	−0.0013 (6)
C6	0.0178 (7)	0.0172 (7)	0.0257 (8)	0.0011 (6)	0.0069 (6)	−0.0006 (6)
C7	0.0201 (7)	0.0191 (7)	0.0201 (7)	−0.0028 (6)	0.0054 (6)	−0.0004 (5)
C8	0.0192 (7)	0.0175 (7)	0.0211 (7)	−0.0023 (6)	0.0044 (6)	0.0028 (5)
C9	0.0198 (7)	0.0160 (7)	0.0197 (7)	−0.0020 (5)	0.0069 (6)	0.0004 (5)
C10	0.0175 (7)	0.0170 (7)	0.0212 (7)	0.0014 (5)	0.0054 (6)	0.0021 (5)
C11	0.0222 (7)	0.0195 (7)	0.0192 (7)	−0.0006 (6)	0.0059 (6)	0.0013 (6)
C12	0.0242 (8)	0.0227 (8)	0.0236 (8)	0.0035 (6)	0.0088 (6)	−0.0016 (6)
C13	0.0216 (7)	0.0199 (8)	0.0259 (8)	0.0026 (6)	0.0061 (6)	0.0019 (6)
C14	0.0394 (10)	0.0229 (9)	0.0378 (10)	0.0001 (7)	0.0165 (8)	−0.0068 (7)
C15	0.0205 (8)	0.0433 (11)	0.0336 (9)	0.0019 (7)	0.0092 (7)	0.0000 (8)
C16	0.0330 (9)	0.0381 (10)	0.0211 (8)	0.0055 (8)	0.0086 (7)	−0.0035 (7)

### Geometric parameters (Å, °)

**Table:**

O1—C6	1.228 (2)	C5—C6	1.512 (2)
O2—C9	1.3733 (19)	C7—C8	1.463 (2)
O2—C14	1.433 (2)	C7—H7A	0.9300
O3—C10	1.3765 (18)	C8—C13	1.395 (2)
O3—C15	1.438 (2)	C8—C9	1.409 (2)
O4—C11	1.3634 (19)	C9—C10	1.397 (2)
O4—C16	1.436 (2)	C10—C11	1.409 (2)
N1—C3	1.335 (2)	C11—C12	1.397 (2)
N1—C2	1.346 (2)	C12—C13	1.387 (2)
N2—C6	1.345 (2)	C12—H12A	0.9300
N2—N3	1.3910 (18)	C13—H13A	0.9300
N2—H1N2	0.91 (3)	C14—H14A	0.9600
N3—C7	1.290 (2)	C14—H14B	0.9600
C1—C5	1.391 (2)	C14—H14C	0.9600
C1—C2	1.394 (2)	C15—H15A	0.9600
C1—H1A	0.9300	C15—H15B	0.9600
C2—H2A	0.9300	C15—H15C	0.9600
C3—C4	1.391 (2)	C16—H16A	0.9600
C3—H3A	0.9300	C16—H16B	0.9600
C4—C5	1.399 (2)	C16—H16C	0.9600
C4—H4A	0.9300		
			
C9—O2—C14	115.72 (13)	O2—C9—C8	118.64 (14)
C10—O3—C15	112.99 (13)	C10—C9—C8	120.32 (14)
C11—O4—C16	117.16 (13)	O3—C10—C9	120.87 (14)
C3—N1—C2	116.26 (15)	O3—C10—C11	119.46 (13)
C6—N2—N3	119.77 (13)	C9—C10—C11	119.68 (14)
C6—N2—H1N2	124.0 (15)	O4—C11—C12	124.39 (15)
N3—N2—H1N2	115.3 (15)	O4—C11—C10	115.49 (14)
C7—N3—N2	112.81 (13)	C12—C11—C10	120.11 (15)
C5—C1—C2	119.00 (16)	C13—C12—C11	119.48 (15)
C5—C1—H1A	120.5	C13—C12—H12A	120.3
C2—C1—H1A	120.5	C11—C12—H12A	120.3
N1—C2—C1	123.62 (16)	C12—C13—C8	121.61 (15)
N1—C2—H2A	118.2	C12—C13—H13A	119.2
C1—C2—H2A	118.2	C8—C13—H13A	119.2
N1—C3—C4	124.95 (16)	O2—C14—H14A	109.5
N1—C3—H3A	117.5	O2—C14—H14B	109.5
C4—C3—H3A	117.5	H14A—C14—H14B	109.5
C3—C4—C5	117.92 (16)	O2—C14—H14C	109.5
C3—C4—H4A	121.0	H14A—C14—H14C	109.5
C5—C4—H4A	121.0	H14B—C14—H14C	109.5
C1—C5—C4	118.23 (15)	O3—C15—H15A	109.5
C1—C5—C6	117.70 (15)	O3—C15—H15B	109.5
C4—C5—C6	124.06 (15)	H15A—C15—H15B	109.5
O1—C6—N2	123.99 (15)	O3—C15—H15C	109.5
O1—C6—C5	121.16 (14)	H15A—C15—H15C	109.5
N2—C6—C5	114.79 (14)	H15B—C15—H15C	109.5
N3—C7—C8	119.94 (14)	O4—C16—H16A	109.5
N3—C7—H7A	120.0	O4—C16—H16B	109.5
C8—C7—H7A	120.0	H16A—C16—H16B	109.5
C13—C8—C9	118.77 (14)	O4—C16—H16C	109.5
C13—C8—C7	121.20 (14)	H16A—C16—H16C	109.5
C9—C8—C7	119.90 (14)	H16B—C16—H16C	109.5
O2—C9—C10	120.87 (13)		
			
C6—N2—N3—C7	−165.58 (14)	C7—C8—C9—O2	7.4 (2)
C3—N1—C2—C1	0.3 (3)	C13—C8—C9—C10	−1.2 (2)
C5—C1—C2—N1	0.5 (3)	C7—C8—C9—C10	−177.24 (14)
C2—N1—C3—C4	−1.0 (3)	C15—O3—C10—C9	82.69 (18)
N1—C3—C4—C5	1.0 (3)	C15—O3—C10—C11	−97.83 (17)
C2—C1—C5—C4	−0.5 (2)	O2—C9—C10—O3	−5.1 (2)
C2—C1—C5—C6	−179.50 (14)	C8—C9—C10—O3	179.63 (14)
C3—C4—C5—C1	−0.2 (2)	O2—C9—C10—C11	175.45 (14)
C3—C4—C5—C6	178.75 (15)	C8—C9—C10—C11	0.2 (2)
N3—N2—C6—O1	7.8 (2)	C16—O4—C11—C12	−3.8 (2)
N3—N2—C6—C5	−175.09 (13)	C16—O4—C11—C10	176.28 (14)
C1—C5—C6—O1	27.6 (2)	O3—C10—C11—O4	1.7 (2)
C4—C5—C6—O1	−151.38 (17)	C9—C10—C11—O4	−178.79 (13)
C1—C5—C6—N2	−149.60 (15)	O3—C10—C11—C12	−178.17 (14)
C4—C5—C6—N2	31.5 (2)	C9—C10—C11—C12	1.3 (2)
N2—N3—C7—C8	−172.80 (13)	O4—C11—C12—C13	178.42 (15)
N3—C7—C8—C13	22.4 (2)	C10—C11—C12—C13	−1.7 (2)
N3—C7—C8—C9	−161.70 (15)	C11—C12—C13—C8	0.6 (2)
C14—O2—C9—C10	73.23 (19)	C9—C8—C13—C12	0.8 (2)
C14—O2—C9—C8	−111.40 (16)	C7—C8—C13—C12	176.81 (15)
C13—C8—C9—O2	−176.62 (14)		

### Hydrogen-bond geometry (Å, °)

**Table:**

Cg1 is centroid of the C1/C2/N1/C3/C4/C5 ring.

**Table:**

*D*—H···*A*	*D*—H	H···*A*	*D*···*A*	*D*—H···*A*
N2—H1N2···O1^i^	0.91 (3)	2.09 (3)	2.7987 (19)	134 (2)
C4—H4A···O1^i^	0.93	2.46	3.348 (2)	159
C14—H14C···O3	0.96	2.55	3.113 (2)	118
C15—H15C···O2^ii^	0.96	2.49	3.292 (2)	141
C16—H16A···O3^iii^	0.96	2.57	3.518 (2)	167
C14—H14B···Cg1^i^	0.96	2.81	3.719 (3)	159

Symmetry codes: (i) −*x*, *y*−1/2, −*z*−1/2; (ii) −*x*+1, −*y*+1, −*z*; (iii) −*x*+1, −*y*+1, −*z*+1.

## References

[bb1] Bruker (2009). *APEX2*, *SAINT* and *SADABS* Bruker AXS Inc., Madison, Wisconsin, USA.

[bb2] Cosier, J. & Glazer, A. M. (1986). *J. Appl. Cryst.* **19**, 105–107.

[bb3] Janin, Y. L. (2007). *Bioorg. Med. Chem.* **15**, 2479–2513. 10.1016/j.bmc.2007.01.030 17291770

[bb4] Kahwa, I. A., Selbin, J., Hsieh, T. C.-Y. & Laine, R. A. (1986). *Inorg. Chim. Acta*, **118**, 179–185.

[bb5] Lourenco, M. C. S., Ferreira, M. L., de Souza, M. V. N., Peralta, M. A., Vasconcelos, T. R. A. & Henriques, M. G. M. O. (2008). *Eur. J. Med. Chem.* **43**, 1344–1347. 10.1016/j.ejmech.2007.08.003 17923172

[bb6] Maccari, R., Ottana, R. & Vigorita, M. G. (2005). *Bioorg. Med. Chem. Lett.* **15**, 2509–2513. 10.1016/j.bmcl.2005.03.065 15863306

[bb7] Naveenkumar, H. S., Sadikun, A., Ibrahim, P., Loh, W.-S. & Fun, H.-K. (2009). *Acta Cryst.* E**65**, o2540–o2541. 10.1107/S1600536809037921 PMC2970303 21577984

[bb8] Naveenkumar, H. S., Sadikun, A., Ibrahim, P., Quah, C. K. & Fun, H.-K. (2010*a*). *Acta Cryst.* E**66**, o291. 10.1107/S1600536810000371 PMC2979869 21579726

[bb9] Naveenkumar, H. S., Sadikun, A., Ibrahim, P., Yeap, C. S. & Fun, H.-K. (2010*b*). *Acta Cryst.* E**66**, o579. 10.1107/S1600536810004514 PMC2983744 21580346

[bb10] Sheldrick, G. M. (2008). *Acta Cryst.* A**64**, 112–122. 10.1107/S0108767307043930 18156677

[bb11] Shi, J. (2005). *Acta Cryst.* E**61**, o3933–o3934.

[bb12] Slayden, R. A. & Barry, C. E. (2000). *Microbes Infect.* **2**, 659–669. 10.1016/s1286-4579(00)00359-2 10884617

[bb13] Spek, A. L. (2009). *Acta Cryst.* D**65**, 148–155. 10.1107/S090744490804362X PMC2631630 19171970

